# Supplementary material for: Cytotoxic Withanolides from the Whole Herb of Physalis angulata L
Source: Molecules. 2019 Apr 23;24(8):1608. doi: 10.3390/molecules24081608 (PMC6514790; doi:10.3390/molecules24081608)
Supplement: Supplementary file 1 [file molecules-24-01608-s001.pdf]

# SUPPLEMENTARY MATERIAL

# Cytotoxic withanolides from the whole herb of *Physalis angulata* L.

Qinghong Meng[1,2,†], JiaJia Fan[2,†], Zhigou Liu[2], Xiwen Li[2], Fangbo Zhang[2], Yanlin Zhang[1,2], Yi Sun[2*], Li Li[3*], Xia Liu[4], and Erbing Hua[1*]

[1] College of Biotechnology, Tianjin University of Science & Technology, Tianjin 300457, China;

[2] Institute of Chinese Materia Medica, China Academy of Chinese Medical Sciences, Beijing100010, China;

[3] Institute of Materia Medica, Chinese Academy of Medical Sciences & Peking Union Medical College, Beijing100010, China;

[4] School of Chemistry, Chemical Engineering and Life Science, Wuhan University of Technology, Wuhan 430070, China.

*Correspondence: Yi Sun, ysun@icmm.ac.cn; Erbing Hua, huarb@ tust.edu.cn; Li Li, annaleelin@imm.ac.cn

† These authors contributed equally to this work.

| Contents | Page No. |
|---|---|
| DNA barcoding identification and picture of the plant | 3 |
| Figure S1: $^{1}$H NMR (600 MHz, DMSO-*D6*) spectrum of **1** | 4 |
| Figure S2: $^{13}$C NMR (150 MHz, DMSO-*D6*) spectrum of **1** | 4 |
| Figure S3: HSQC spectrum of **1** | 5 |
| Figure S4: HMBC spectrum of **1** | 5 |
| Figure S5: $^{1}$H-$^{1}$H COSY spectrum of **1** | 6 |
| Figure S6: NOESY spectrum of **1** | 6 |
| Figure S7:CD spectrum of **1** | 7 |
| Figure S8: $^{1}$H NMR (600 MHz, DMSO-*D6*) spectrum of **2** | 7 |
| Figure S9: $^{13}$C NMR (150 MHz, DMSO-*D6*) spectrum of **2** | 8 |
| Figure S10: HSQC spectrum of **2** | 8 |
| Figure S11: HMBC spectrum of **2** | 9 |
| Figure S12: $^{1}$H-$^{1}$H COSY spectrum of **2** | 9 |
| Figure S13: NOESY spectrum of **2** | 10 |
| Figure S14: CD spectrum of **2** | 10 |

Identification results of DNA barcoding:

The similarity search by the NCBI gene database indicated that the species was *Physalis angulata* L., which is consistent with the identification of the identification experts. The ITS2 sequence peak map and sequence results are as follows:

>*Physalis angulata*_ITS2

CGCATCGCGTCGCCCCCCTCGCCCCGCACTGCGGGGCGTCGCGGGACGGATACTGGCC

TCCCGTGCGCTCTCAGCGCGCGGCTGGCCTAAATGCGAGCCCACGTCGACGGACGTCA

CGGCAAGTGGTGGTTGAATCTCAACTCTCTTGGTGCCGTGGCCGAACCCGTCGCCCGT

GTCGGCTGCGAGACCCTTCCGGCGCTCTGGCGCTCCGACCG

The picture of the sample of *Physalis angulata*:

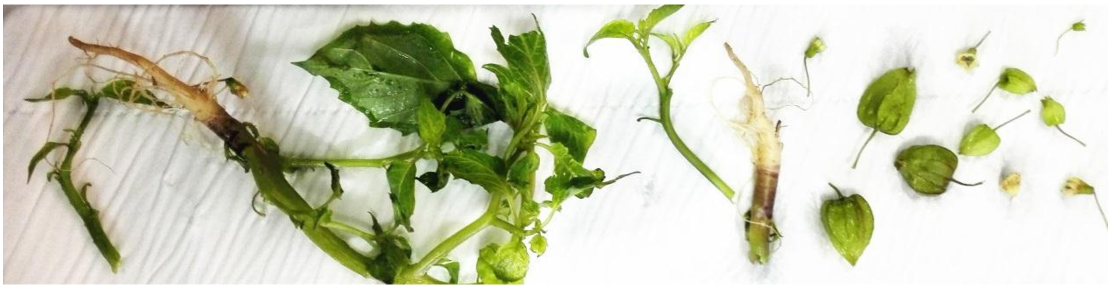

Figure S1: $^{1}$H NMR (600 MHz, DMSO-*D6*) spectrum of **1**

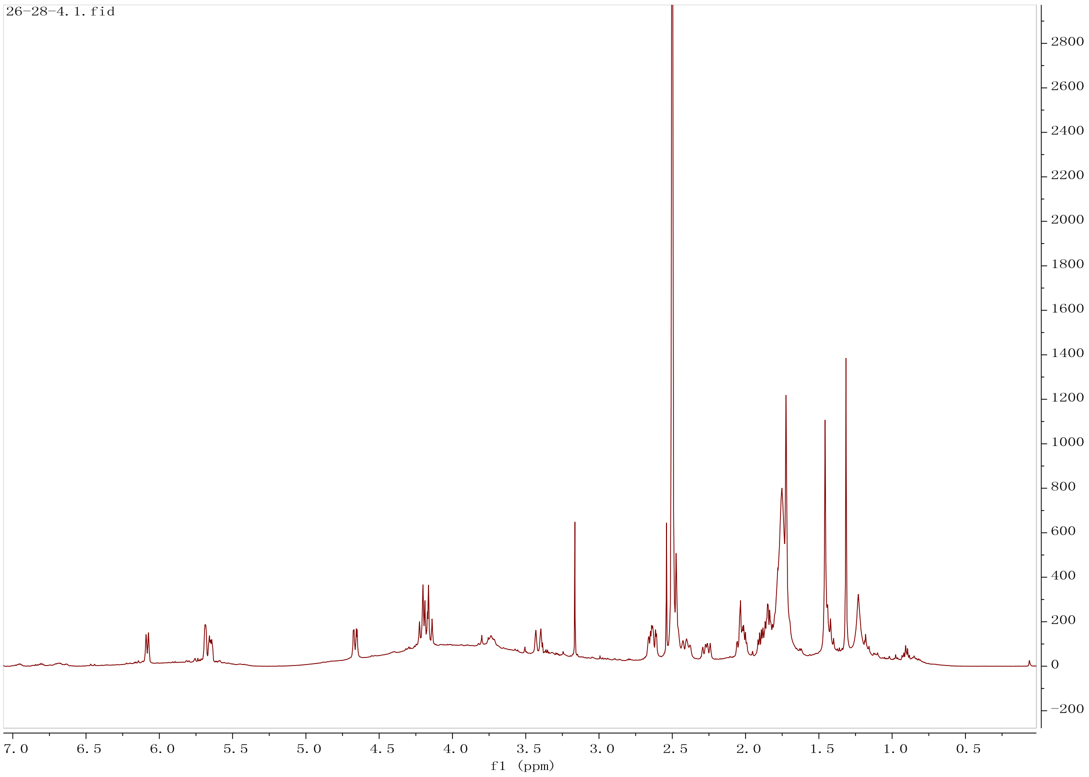


Figure S2: $^{13}$C NMR (150 MHz, DMSO-*D6*) spectrum of **1**

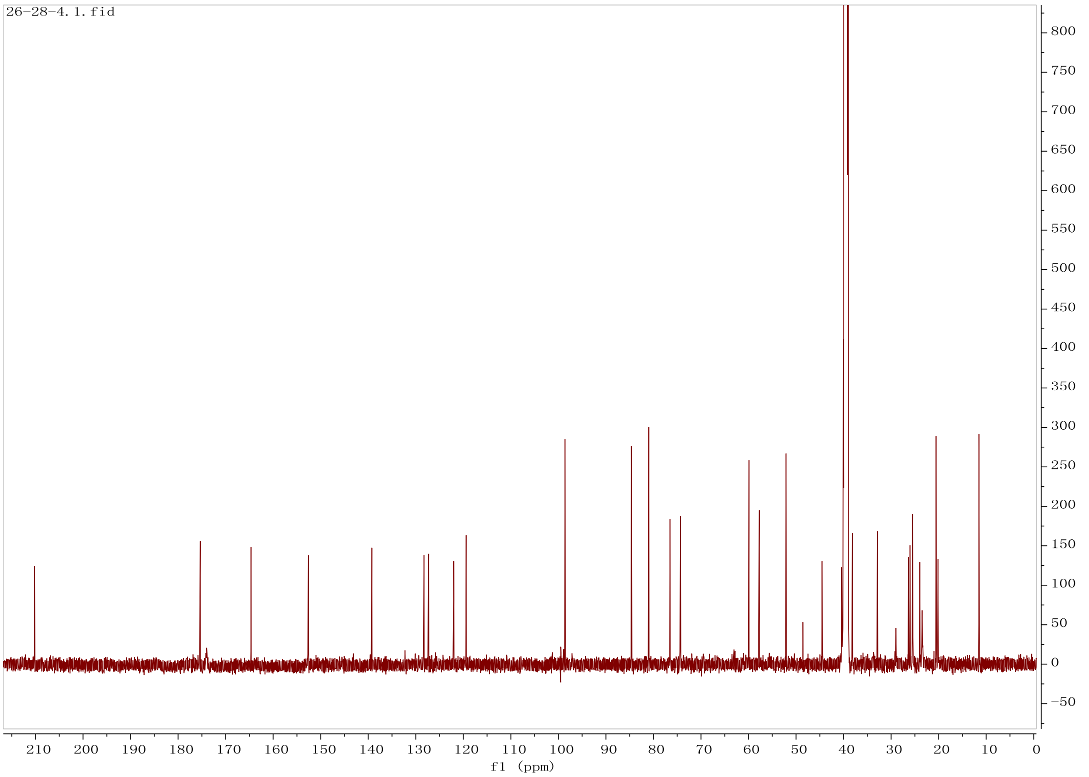

Figure S3: HSQC spectrum of **1**

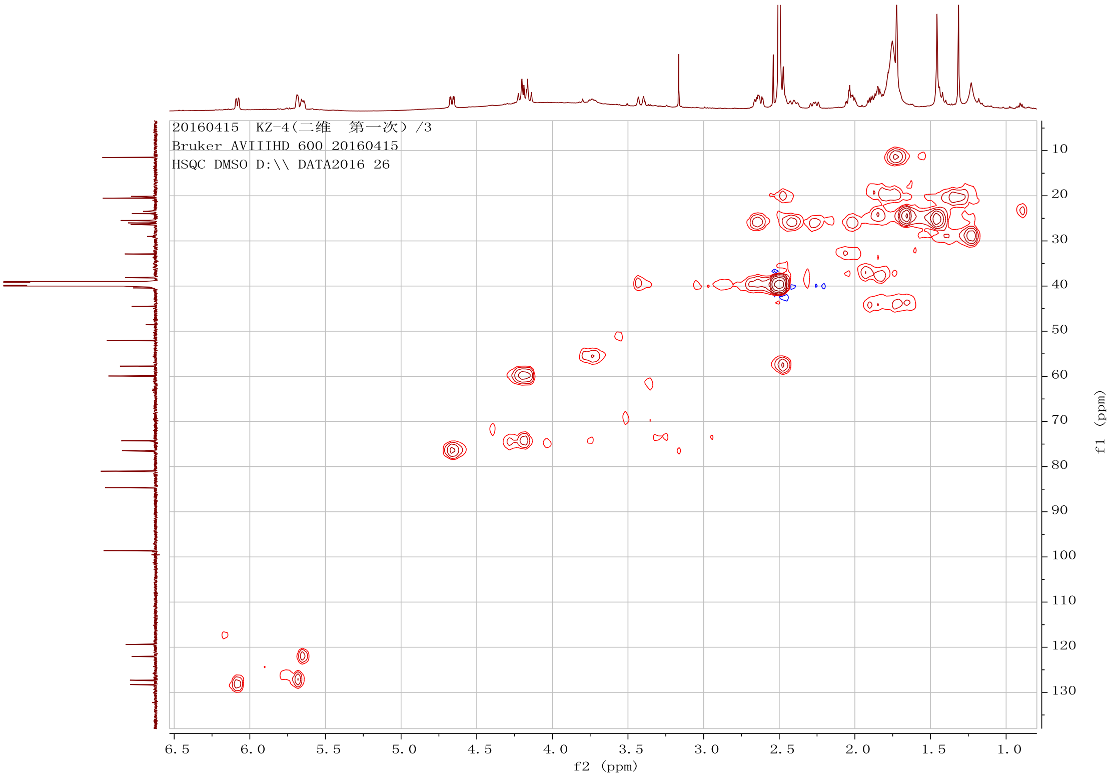


Figure S4: HMBC spectrum of **1**

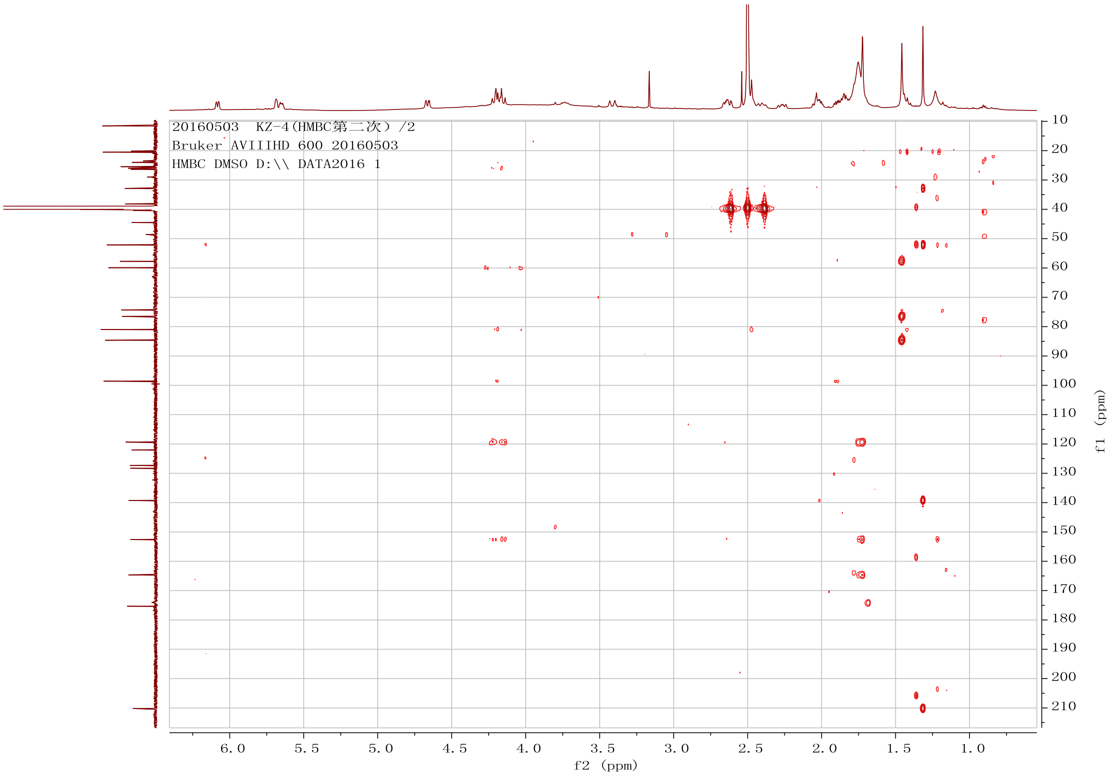

Figure S5: $^{1}H$-$^{1}H$ COSY spectrum of **1**

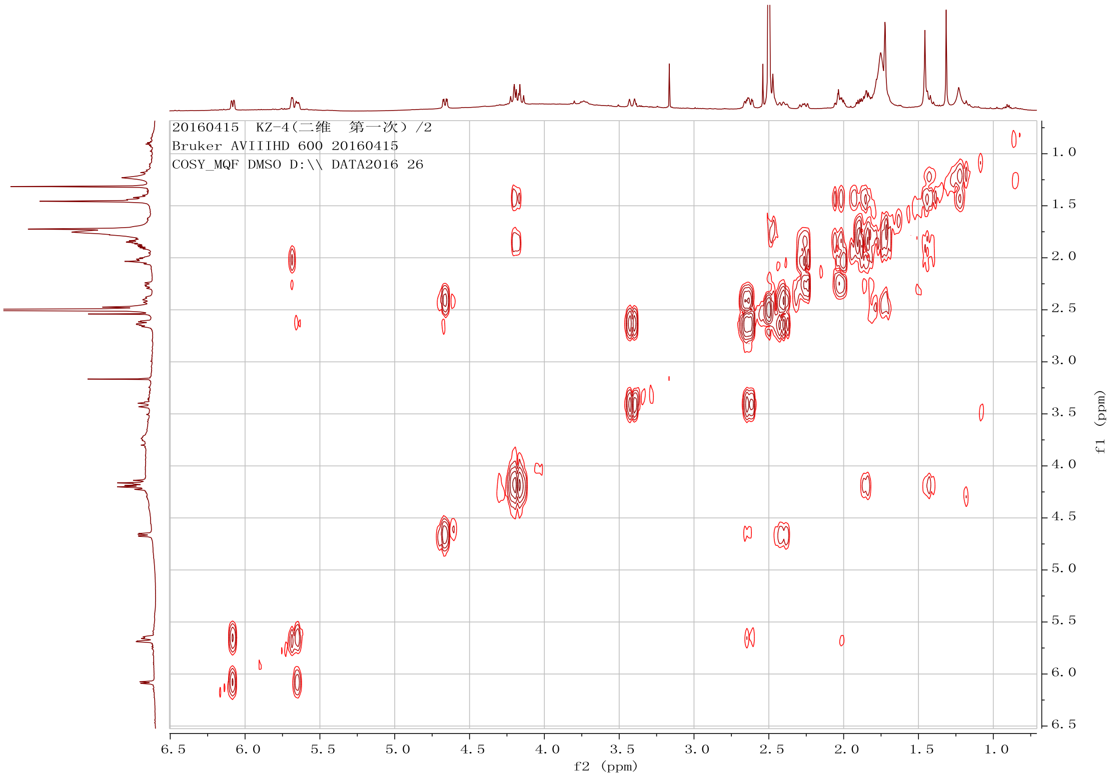


Figure S6: NOESY spectrum of **1**

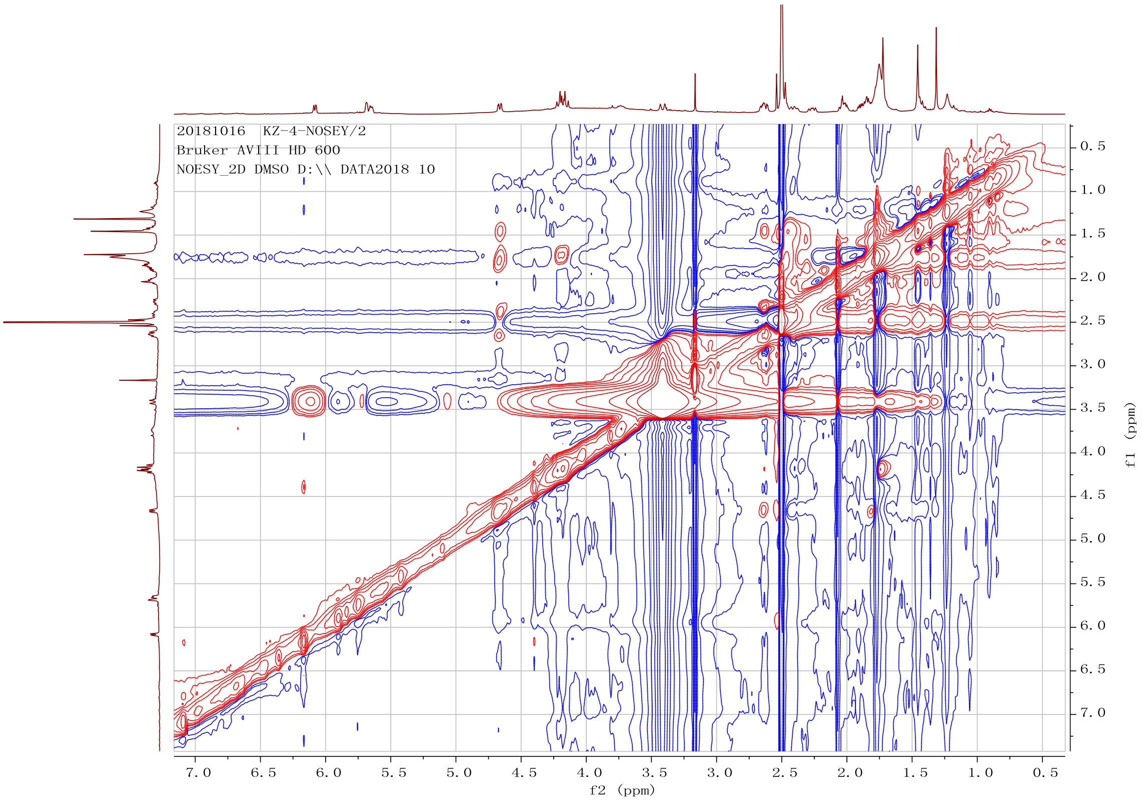

Figure S7: CD spectrum of **1**

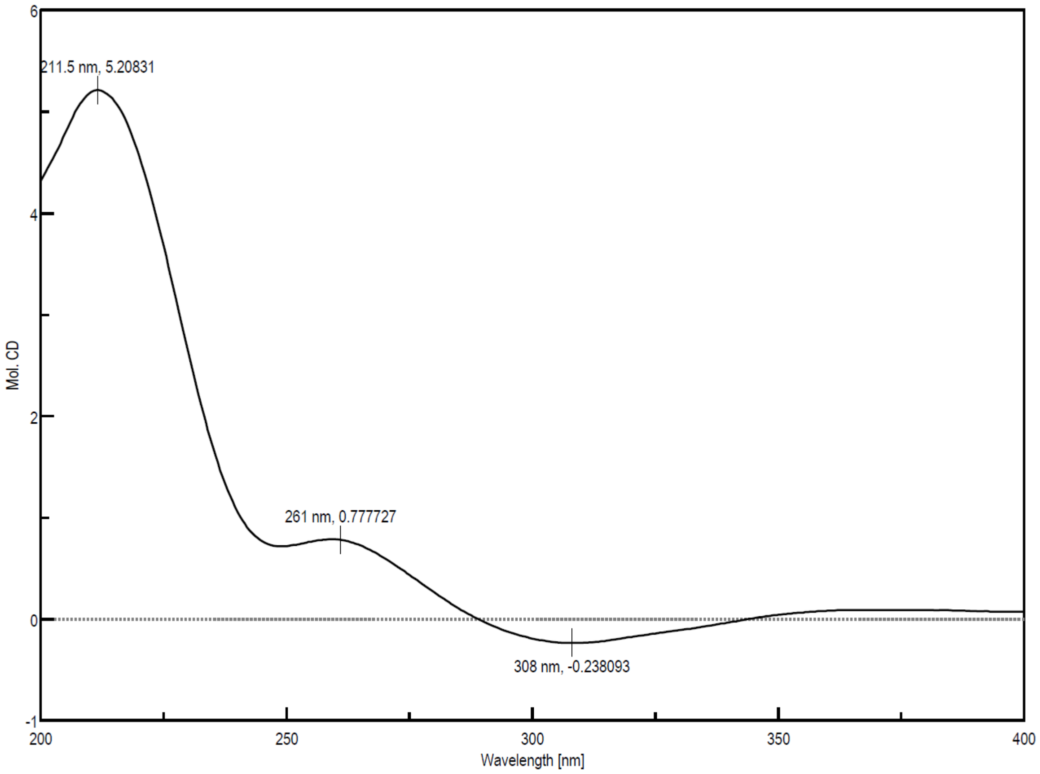


Figure S8: $^{1}$H NMR (600 MHz, DMSO-*D6*) spectrum of compound **2**

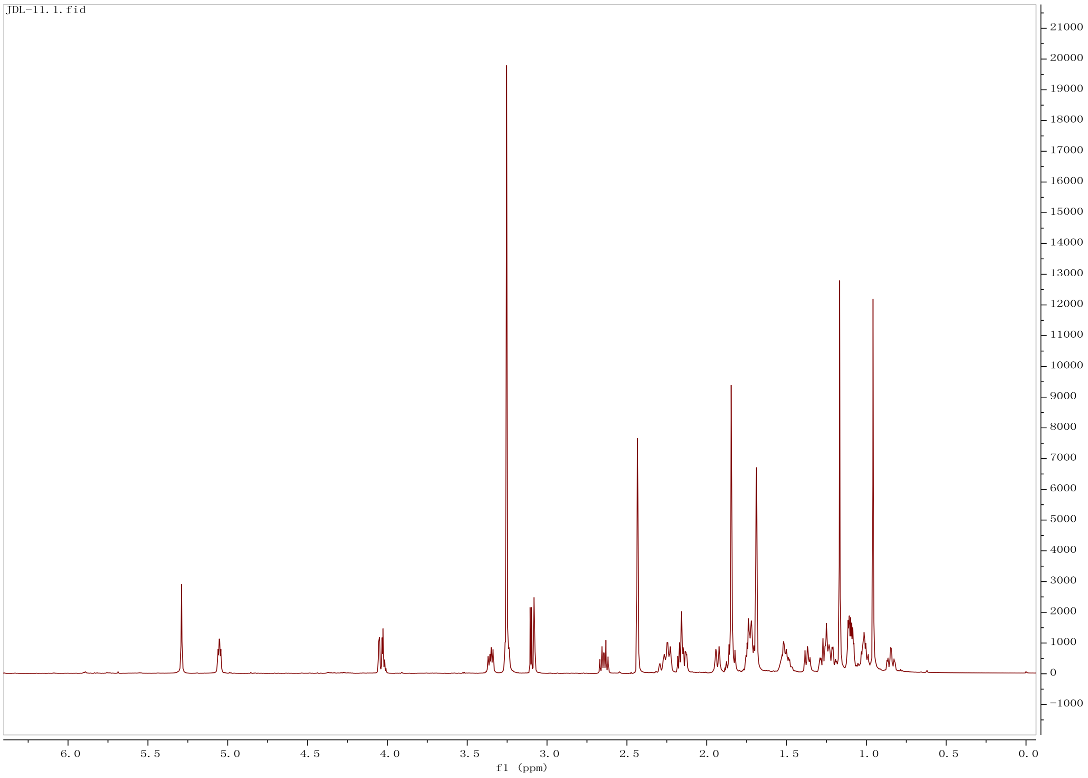

Figure S9: $^{13}$C NMR (150 MHz, DMSO-*D6*) spectrum of **2**

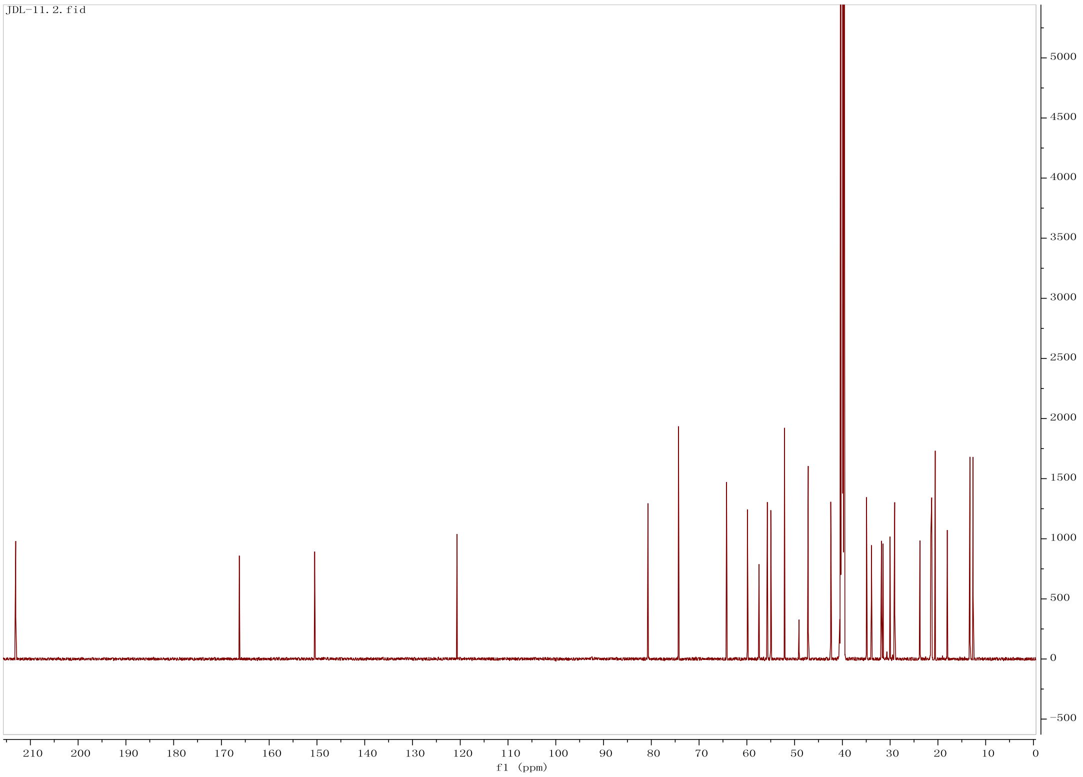


Figure S10: HSQC spectrum of **2**

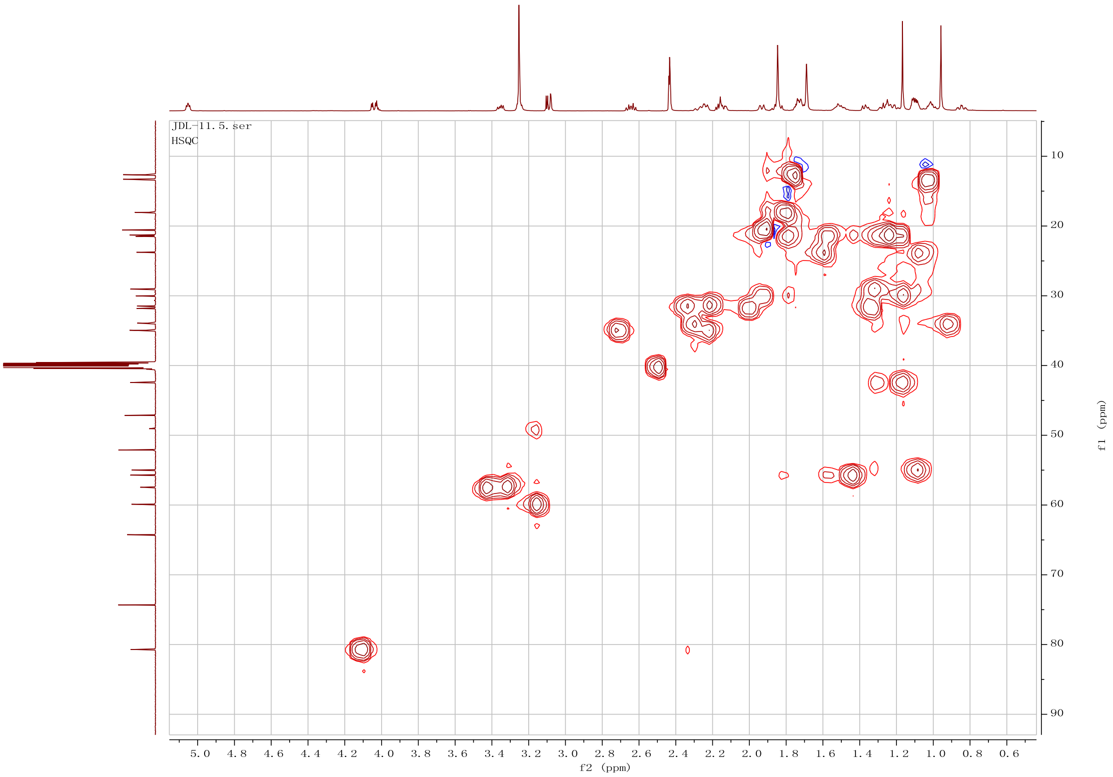

Figure S11: HMBC spectrum of **2**

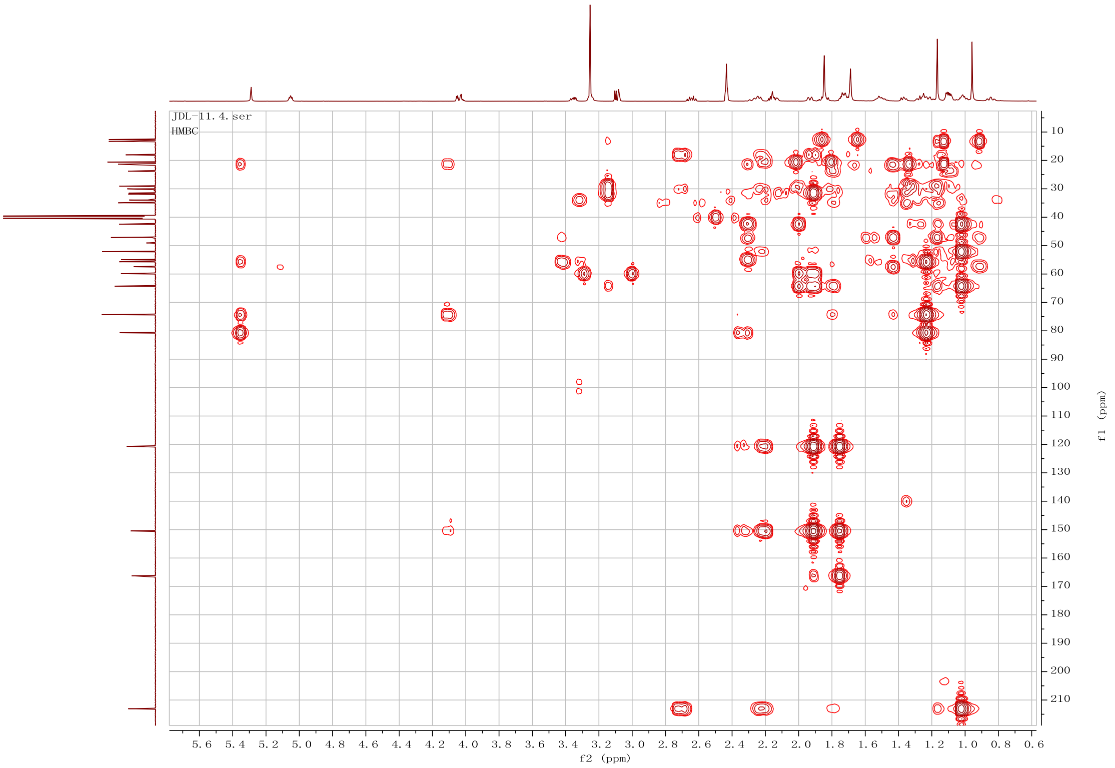


Figure S12: $^{1}H$-$^{1}H$ COSY spectrum of **2**

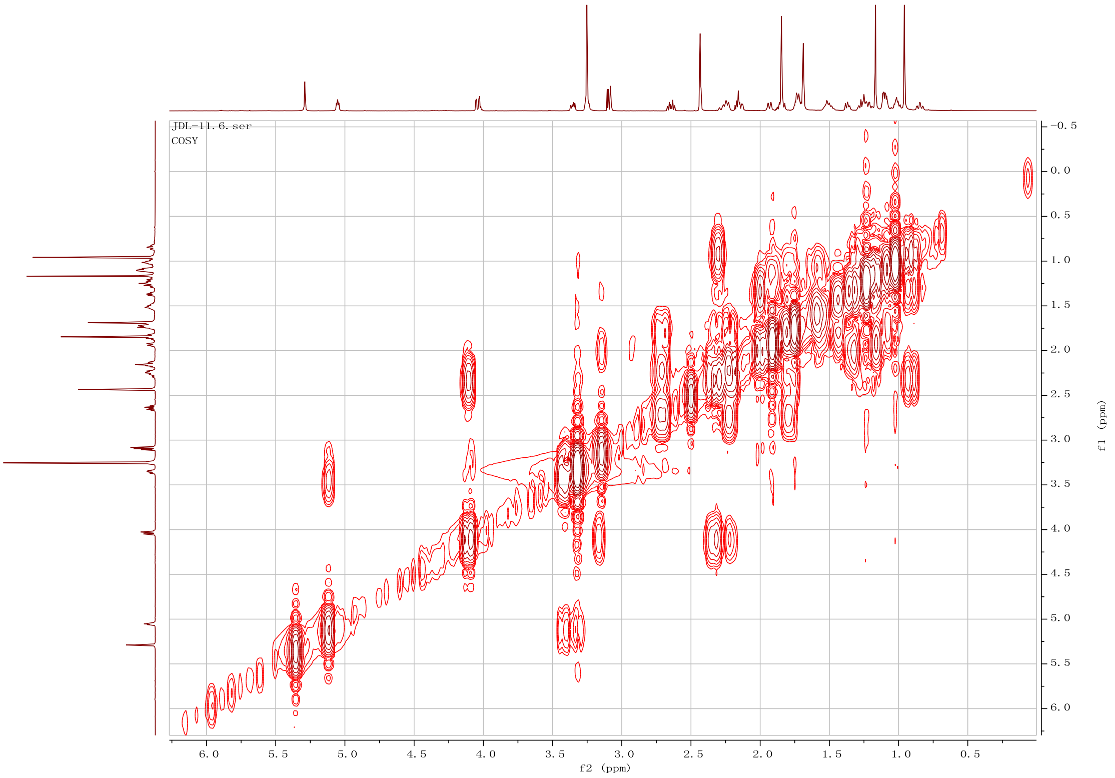

Figure S13: NOESY spectrum of **2**

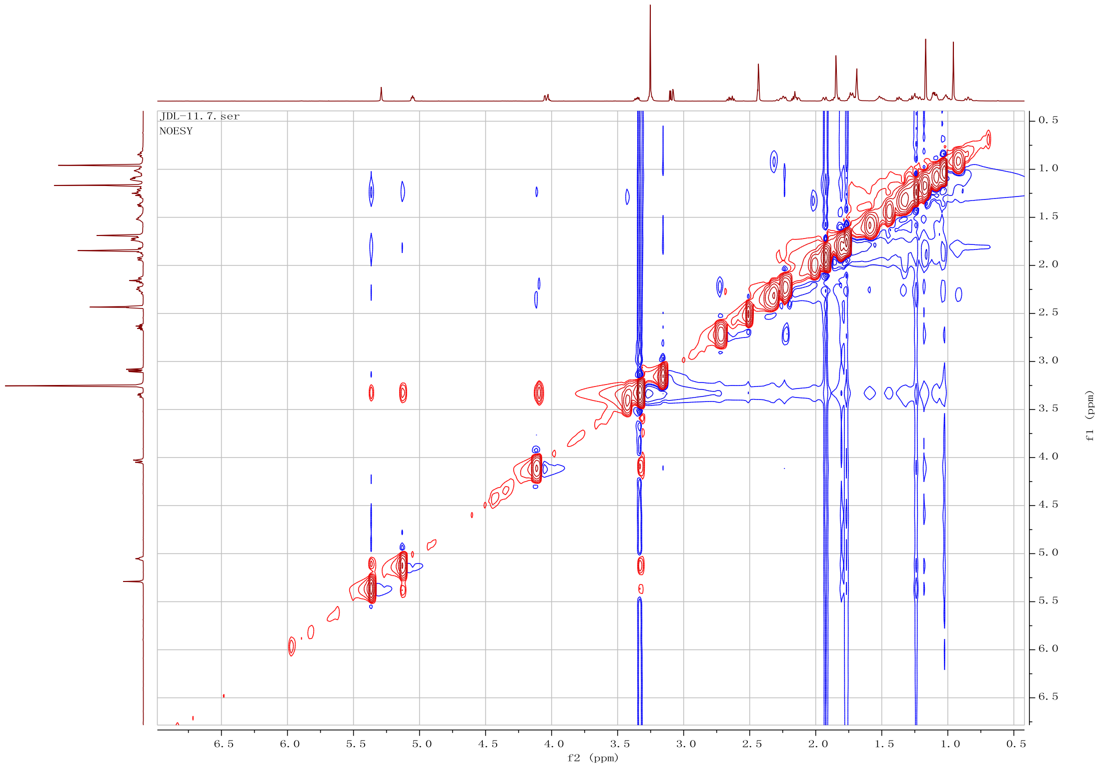


Figure S14: CD spectrum of **2**

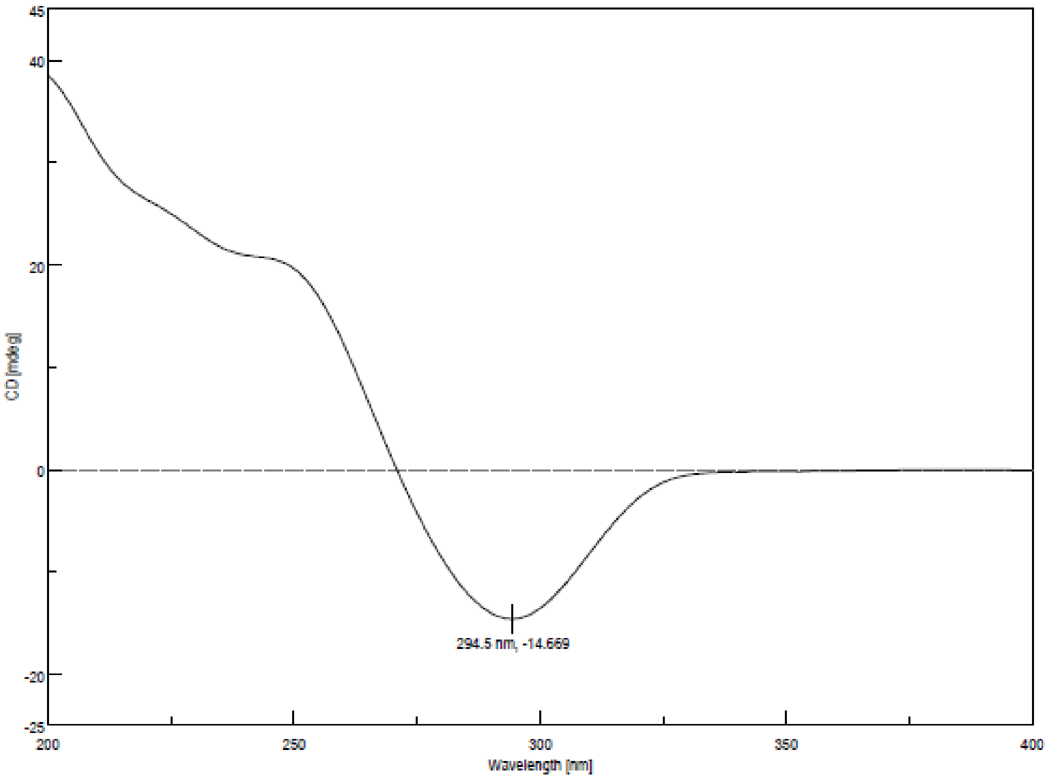

Identification results of DNA barcoding:

The similarity search by the NCBI gene database indicated that the species was *Physalis angulata* L., which is consistent with the identification of the identification experts. The ITS2 sequence peak map and sequence results are as follows:

>*Physalis angulata*_ITS2

CGCATCGCGTCGCCCCCCTCGCCCCGCACTGCGGGGCGTCGCGGGACGGATACTGGCC

TCCCGTGCGCTCTCAGCGCGCGGCTGGCCTAAATGCGAGCCCACGTCGACGGACGTCA

CGGCAAGTGGTGGTTGAATCTCAACTCTCTTGGTGCCGTGGCCGAACCCGTCGCCCGT

GTCGGCTGCGAGACCCTTCCGGCGCTCTGGCGCTCCGACCG
